# Highlights from the tenth ISCB Student Council Symposium 2014

**DOI:** 10.1186/1471-2105-16-S2-A1

**Published:** 2015-01-28

**Authors:** Farzana Rahman, Katie Wilkins, Annika Jacobsen, Alexander Junge, Esmeralda Vicedo, Dan DeBlasio, Anupama Jigisha, Tomás Di Domenico

**Affiliations:** 1 Genomics and Computational Biology Research Group, University of South Wales, UK; 2 Computational Biology Department, Cornell University, USA; 3 Centre for Integrative Bioinformatics (IBIVU), VU University Amsterdam, Netherlands; 4 Center for non-coding RNA in Technology and Health, University of Copenhagen, Denmark; 5 Department of Bioinformatics and Computational Biology, Fakultät für Informatik, Germany; 6 Department of Computer Science, University of Arizona, USA; 7 University of Geneva, Switzerland; 8 Wellcome Trust/Cancer Research UK Gurdon Institute, University of Cambridge, UK

## Abstract

This report summarizes the scientific content and activities of the annual symposium organized by the Student Council of the International Society for Computational Biology (ISCB), held in conjunction with the Intelligent Systems for Molecular Biology (ISMB) conference in Boston, USA, on July 11^th^, 2014.

## About the Student Council and the symposium

The Student Council (SC), part of the International Society for Computational Biology (ISCB), aims at nurturing and assisting the next generation of computational biologists. Our membership and leadership are composed of volunteer students and post-docs in computational biology and related fields. The main goal of our organisation is to offer networking and soft skill development opportunities to our members.

The Student Council Symposium (SCS) takes place every year, directly preceding the ISMB/ECCB conferences. SCS 2014 marked the tenth consecutive edition of the event, after the success of previous years' editions [1-7].

## Meeting format

The Student Council Symposium is a one-day event. Following the success of previous years, SCS 2014 kicked off with a scientific speed dating session. During this session our delegates have to find a partner to introduce themselves to, and they discuss their scientific backgrounds and interests. After five minutes they must switch partners, and this goes on until the allotted time runs out. The traditional scientific component of the meeting consisted of two keynote presentations by senior researchers, twelve oral presentations by delegates, and a poster session.

To celebrate the 10^th ^edition of the Student Council Symposium, Dr. Manuel Corpas, Dr. Jeroen DeRidder, Dr. Nils Gehlenborg and Dr. Geoff Macintyre, former members of the Student Council, delivered a welcome address and an overview of the Student Council's history.

Dr. David Bartel (HHMI/MIT/Whitehead Institute, US) and Dr. Ashlee Earl (The Broad Institute of MIT & Harvard, US) generously agreed to deliver the keynote addresses at SCS 2014. In addition, Abhishek Pratap, Senior Research Scientist in Bioinformatics at our institutional partner Sage Bionetworks, gave a short presentation about Enabling Collaborative and Reproducible Research through the Synapse software.

SCS 2014 received 76 submissions from students, which were peer-reviewed by 23 independent reviewers. More than 50 abstracts were accepted for poster presentations, and 12 abstracts were invited to deliver an oral presentation. Extended abstracts of oral presentations are included in this report. All abstracts are available online in the SCS 2014 booklet http://scs2014.iscbsc.org/booklet-2014.

## Welcome address: 10 years of Student Council

The commemorative welcome address opened the day, and Drs. Corpas, DeRidder, Gehlenborg and Macintyre provided our delegates with their points of view on the evolution of the Student Council during its first 10 years. Having now become young group leaders and senior postdocs, they offered an interesting perspective on the impact the Student Council has had on the development of their carreers.

## Keynotes

Dr. David Bartel's keynote followed the welcome address. In his talk, Dr. Bartel gave an overview of the current understanding of microRNAs, the progress in predicting their targets, and how measurements of their regulatory effects have revealed an unexpected developmental switch in the nature of mRNA translational control.

Dr. Ashlee Earl gave us an overview of her work on tackling longstanding and emerging challenges in infectious disease by taking advantage of new sequencing technologies. In particular, she described her group's work on tackling the emergence of multi-drug resistant strains of pathogens through the development of approaches and tools to examine the drug resistant Mycobacterium tuberculosis.

## Student presentations

The first oral presentation was delivered by Yassine Soulimi, who introduced the COSMOS software for cloud enabled next generation sequencing analysis [8]. COSMOS is a scalable workflow management framerwork, which aims at reducing the cost of whole genome data analysis in order to place it within a reimbursable cost point and in clinical time.

When performing multiple sequence alignments, most users tend to rely on the default parameters of the algorithm. A different parameter setting may however have great impact on the quality of the output alignment. Parameter advising is the task of selecting good parameters for a given set of input sequences to be aligned. Dan DeBlasio presented his work on constructing improved advisors for multiple sequence alignment [9].

Lin-Yang Cheng described his efforts to enhance quantitative protein-level conclusions in experiments with data-independent spectral acquisition by the statistical elimination of spectral features with large between-run variation. His results show that his approach achieves an accuracy that exceeds the standard approach of using three spectral features with the highest intensity between runs [10].

Microsatellites are short, tandem-repeated DNA sequences which make up approximately 3% of the human genome. The expansion of these microsatellite repeats has been linked to many neurological and developmental disorders. Harriet Dashnow presented her work on developing a microsatellite genotyping algorithm that addresses several issues regarding the length determination of microsatellites from next-generation sequencing data, and provides a highly accurate and more detailed analysis of microsatellite loci [11].

Yi Zhong reported the development of novel computational tools to gain biological insight from Ribo-seq and RNA-seq data in a fast and accurate way. By using transcriptome-scale ribosome footprinting data from leukemia cell lines, he identified drug-sensitive genes showing both decrease of translational efficiency and accumulation of ribosome occupancy at 5'UTRs. These genes constitute potential therapeutic targets for cancer [12].

The usage of tetranucleotides for genomes analysis is a promising approach for the evaluation of host-parasite coevolution and gene exchange within the mycobacteriophage population. Benjamin Siranosian talked about computationally inexpensive methods, based on the usage of tetranucleotides, that are also independent of gene annotation, and their usefulness for phage clustering and the analysis of evolutionary relationships [13].

Haeewook Lee presented his work on the detection of structural variants involving insertion sequence elements in mutation accumulation lines of Escherichia coli. By extending an A-Bruijn graph based structural variant detection framework he was able to tackle the challenge of obtaining direct estimates on insertion, deletion and recombination event rates.

Using a Random Forest machine learning approach, Russel Sutherland reported results on the discrimination between cancer differentiation subtypes. By applying the algorithm to exome sequencing data from tumour and normal tissue samples from 1798 patients, they were able to discriminate between 5 cancer types with high accuracy.

Alex Salazar presented Emu, an algorithm that resolves alternate representations of larger sequence variants (LSVs) by comparing variants across genomes. Emu improves the analysis of LSVs in bacterial genomes by reducing cross-sample noise resulting from per-sample variant calls [14].

Vikas Pejaver presented MutPred2, a method for the prediction of pathogenicity of missense variants and their molecular effects. The software can be used to guide downstream experiments for elucidating the molecular basis of disease, and to assist in the development of therapeutic strategies.

Sarah Keasey presented her work on systematically identifying and analysing thousands of direct binary protein interactions within *Y. pestis*. The resulting benchmark dataset can be highly useful for the analysis of protein interaction networks functioning within an important human pathogen [15].

On behalf of Amin Ardeshirdavani, Prof. Yves Moreau presented NGS-Logistics, a platform to analyse NGS data in a distributed way, while guaranteeing privacy and security [16]. The framework aims to reduce the effort and time needed to evaluate the significance of mutations based on full genome and full exome sequencing.

## Award winners

Thanks to the generous contribution of the Swiss Institute of Bioinformatics, two travel fellowships were awarded to Sarah Keasey and Vikas Pejaver to attend SCS 2014.

Based on the votes of the SCS delegates, a judging committee awarded three speakers with one best oral and two best poster presentations awards. The best oral presentation award went to Harriet Dashnow for her work entitled "Genotyping Microsatellites in Next-Generation Sequencing Data". The first place in the best poster presentation awards went to Alex Salazar for his work, "Investigating large sequence variants in drug resistant Mycobacterium tuberculosis". The second place in the poster presentation awards went to Sarah Keasey for her work, "The Road To Linking Genomics And Proteomics Of Pathogenic Bacteria: From Binary Protein Complexes To Interaction Pathways".

In addition to the aforementioned awards, Russell Sutherland and Dilmi Perera received F1000 awards for their poster presentations at SCS 2014.

## Conclusions

This year's number of submissions and participants saw a slight decline in comparison with the previous edition. Visa issues and the general lack of funding seem to be the main reasons according to our surveys. All these issues notwithstanding, the quality of the keynote presentations, the 12 oral presentations and the poster session once again made the Student Council Symposium a great success.

Preparations are already ongoing for the 11^th ^edition of SCS to be held in Dublin, Ireland, preceding ISMB/ECCB 2015. For further information regarding the Student Council, its events, internships and community, please visit http://www.iscbsc.org.
